# miR-877-3p inhibits tumor growth and angiogenesis of osteosarcoma through fibroblast growth factor 2 signaling

**DOI:** 10.1080/21655979.2021.1982305

**Published:** 2022-03-21

**Authors:** Mingji Chen, Zhi Li, Lei cao, Chi Fang, Rufeng Gao, Chao Liu

**Affiliations:** a Department of Orthopedics, Qingpu Branch of Zhongshan Hospital, Fudan University, Shanghai, China; b Department of Orthopedics, Shanghai Songjiang District Central Hospital, Shanghai, China; c Department of Radiology, Shanghai Songjiang District Central Hospital, Shanghai, China; d Department of Gynecologic Oncology, Fudan University, Shanghai Cancer Center, Shanghai, China

**Keywords:** Mir-877-3p, osteosarcoma, proliferation, angiogenesis, fgf2

## Abstract

Osteosarcoma (OS) is the most common high-grade malignant bone tumor in teenagers. MicroRNAs can function as posttranscriptional regulators of gene expression, playing critical roles in cancer dev-877-3p in OS. Quantitative real-time RT-PCR was carried out for detecting miR-877-3p expression in OS. The effects of miR-877-3p on proliferation was analyzed via MTT, colony formation, and flow cytometry assays. Angiogenesis of endothelial cells were investigated by wound healing and tube formation assay. Gene profiling based on PCR array and luciferase reporter assay were conducted to determine target genes of miR-877-3p. In-vivo study was used to determine the effects of miR-877-3p on the tumor growth. The expression of miR-877-3p was markedly downregulated in OS tissues and cell lines. Low expression of miR-877-3p predicts poor prognosis of OS patients. miR-877-3p overexpression was found to inhibit the proliferation of OS cell lines. The angiogenesis assays showed that miR-877-3p attenuated the angiogenesis. Further mechanism studies showed that miR-877-3p can reduce (Fibroblast Growth Factor 2) FGF2 expression in OS cells by binding to the 3’UTR end of FGF2. Moreover, increased expression of miR-877-3p was responsible for the inhibition of tumor growth and angiogenesis. Taken together, our findings indicated that miR-877-3p might exhibit tumor suppressive role by targeting FGF2 signaling, which may serve as potential target for OS.

## Introduction

Osteosarcoma (OS) is one of the most prevalent type of primary malignant bone tumor that occurs in teenagers or children under 20 years old [1]. Although Great efforts have been made in OS treatment, no significant improvement of survival has been achieved for decades. The etiology and pathogenesis are not completely clear until now. However, genetic and epigenetic factors were thought to may play a key role in OS tumorigenesis [2]. Thus, discovering the underlying molecular mechanisms are urgently needed in OS.

MicroRNAs are a class of short (20–30 nucleotide) RNA molecules that regulate posttranscriptional gene expression by binding to the 3-untranslated region (3ʹ-UTR) of the protein-coding messenger RNA (mRNA) [3]. MicroRNAs have been reported to play crucial roles in various biological processes, and abnormal microRNA expression is related to the impairment of normal biological function [4]. Some studies have demonstrated that microRNAs are involved in the tumorigenesis of osteosarcoma, indicating its potential in OS diagnostics and therapeutics [5–7].

Angiogenesis is the process of new blood vessel development, which is critical in tumor progression. OS is characterized by intensive vascularity with a high potency to form visceral metastases. Therefore, antiangiogenesis therapy in OS has achieved more attention [8,9]. Fibroblast growth factor receptor (FGFR) signaling, which is related to angiogenesis, plays a comprehensive role in many physiological and pathological processes. FGF2, or named as basic FGF (bFGF), is the most studied member of the FGF superfamily, and was found to can promote OS progression [10]. FGF2 is also highly expressed in OS environment, indicating an importance role of FGF2 in OS angiogenesis.

Herein, we sought to explore the role of miR‐877-3p in OS cells proliferation and vascular endothelial cell line angiogenesis process. The miR‐877-3p target genes of were also investigated to explore the regulatory mechanism of miR‐877-3p. We hypothesized that miR‐877-3p may exert suppressive effects on OS cells and vascular endothelial cell by targeting certain downstream mRNA. The study may provide a new potential target for the clinical diagnosis and treatment of OS in the future.

## Materials and methods

### Public dataset extraction and analysis

MicroRNA expression profiles were obtained from the Gene Expression Omnibus (GEO) (http://www.ncbi.nlm.nih.gov/geo/). MicroRNA expression data set based on GPL19631 Exiqon human V3 microRNA PCR panel I+ II was deposited by Allen et al with the accession number of GSE65071 [11], which contains 20 human OS tumor tissues and 15 control tissues. R software version 3.5.2 obtained from https://www.r-project.org and the Bioconductor package ‘Limma’ v3.36.5 (https://bioconductor.org/packages/limma/) were used for analyzing raw data of the gene expression profiles. Differentially expressed genes (DEGs) between tumor and normal tissues were screened with ‘Limma’ package. The screening criteria for DEGs were both adjusted to *P* < 0.01 and log_2_ fold change > 2.0. Fold change value of differentially expressed microRNAs were plotted.

### Patients, tissue samples and cell lines

In the present study, we used 71 pairs of primary OS and matched adjacent normal bone tissues which were located 3 cm away from the tumor margin from the patients who underwent tumor resection at the Institute of Shanghai Songjiang Central Hospital between August 2013 and January 2018. Overall survival (OS) was estimated from the date of surgery to the date of death from any cause or last follow-up. At recruitment, the written informed consent was signed and obtained from each participant. This study was approved by the ethical review board of the Institute of Fudan University Shanghai Cancer Center and adhered to Declaration of Helsinki. The reference number is 050432-4-1805 C. 

Human umbilical vein endothelial cells (HUVEC) and OS cell lines (Saos2, SJSA-1, U2OS, and MG63) were purchased from American Type Culture Collection (ATCC, USA). Normal human osteoblast cell (NHOst) was obtained from lab stock. All cells were cultured in RPMI1640 medium (Invitrogen, USA) supplemented with 10% FBS (Gibco, USA) in a 37°C humidified incubator containing 5% CO2 as previously described [12,13].

### RNA extraction and SYBR green quantitative PCR analysis

Total RNA was extracted from cells with Trizol reagent (Invitrogen). The abundance of miR-877-3p was measured using TaqMan miRNA assays (Applied Biosystems, USA) in accordance with the manufacturer’s protocols. U6 was used as endogenous control. Reverse transcription was performed using PrimeScript™ RT reagent kit (TakaRa, Japan). FGF2 expression was qualified using SYBR Premix Ex Taq II (Takara, Japan) and normalized with housekeeping gene β-actin. The primers were as follows: β‐actin: CTGGAACGGTGAAGGTGACA (forward), CGGCCACATTGTGAACTTTG (reverse); FGF2: TTCTTCCTGCGCATCCAC (forward), CCTCTCTCTTCTGCTTGAAGTTG (reverse). All expression data were processed using the 2^−ΔΔCT^ method [14].

### Human angiogenesis RT^2^ Profiler PCR Array

The cDNA of HUVEC transfected with miR-877-3p mimics or negative control were synthesized by the RT^2^ first strand kit according to previous study [15] and manufacturer's protocol (Qiagen, USA) . Gene expression profiling was performed using the RT^2^ profiler PCR array with 84 angiogenesis pathway genes (PAHS-024Z, Qiagen, USA). Quantitative reverse transcriptase PCR was conducted using 7500 Real Time PCR System (AB Applied Biosystems, Germany). Expression data were processed using the 2^−ΔΔCT^ method.

### In vitro tube formation assays

Tube formation assay was performed to analyze cellular angiogenic ability [13]. Briefly, a 96-well plate was precoated with Matrigel (BD Pharmingen, USA) overnight. HUVECs transfected with miR‐877-3p mimics or control oligonucleotides were seeded into the plate at the density of 2 × 10^4^ cells/well incubation for 12 h. The capillary-like structures were photographed with microscope.

### Cell transfection

The miR-877-3p mimics and negative control were purchased from Guangzhou RiboBio Co., Ltd. (Guangzhou, China). FGF2 overexpression plasmid and the control plasmid were purchased from Sino Biological Co., Ltd (Beijing, China). Cell transfection was performed as described previously [16]. In brief, cells were seeded in 60 mm plates 1 day before transfection. Cells were transfected with miR-877-3p mimics or NC or plasmids by Lipofectamine 2000 (Invitrogen, USA) using serum-free medium. After 5 h of transfection, the cells were seeded in complete medium and cultured for further experiments.

### Cell viability assays

MTT assay was performed as described before [17]. Cells were plated out at a concentration of 103 cells per well in 96-well plate and maintained at 37°C for 2, 3, 4, 5, 6, or 7 days after transfection, then incubated with 0.5 mg/ml 3-(4,5-dimethylthiazol-2-yl)-2,5-diphenyltetrazolium bromide (MTT, Sigma) at 37°C for 4 h. Optical density (OD) value of cells at 570 nm was measured by a microplate reader.

### Wound healing assays

Wound healing assays of HUVEC cells were conducted according to previous study [18]. HUVEC cells were seeded at 2 × 105 cells/well in a 6-well plate until reached 70–80% confluence. Afterward, the monolayer was scratched using a 100-μl sterile. Then cells were washed with PBS three times to remove debris and were incubated in medium contained low FBS for 0 or 36 h. Then, the images were captured and migration rate was measured under the microscope .

### Colony formation assays

Colony formation assays assay was performed as described previously [19]. To evaluate the clonogenic ability, 400 OS cells were incubated in 6-well culture plates for 2 weeks. Colonies were stained by crystal violet (Sigma, USA).

### Flow cytometric analysis

Cell cycle was evaluated using flow cytometry [20]. About 48 h after transfection, cells were harvested, and were washed to single-cell suspension with cold PBS, and were fixed with 75% ethanol overnight at 4°C. Then, the fixed cells were incubated with 0.01% RNase at 37°C for 10 min and subjected to propidium iodide (PI) at 4°C for 20 min, followed by flow cytometric analysis using BD Accuri® C6 analyzer. The results were immediately analyzed using FlowJo software.

### Dual-luciferase assay

Luciferase reporter assay was performed as shown before [21]. The wild type (FGF2-3’-UTR-WT) or mutant (FGF2-3’-UTR-MUT) 3’-UTR region of FGF2, which were predicted to interact with miR-877-3p, were respectively cloned into pGL3 luciferase vector. The indicated cells were seeded into a 96-well plate. After 24 h, FGF2-3’-UTR-WT and miR877-3p mimics or FGF2-3’-UTR-MUT and miR-877-3p mimics were transfected into the treated OS cells or HUVEC using Lipofectamine 2000 (Invitrogen, USA). After 48 h transfection, relative luciferase activity was detected in cell lysates by the Dual-Luciferase Reporter Assay System (Promega, USA) according to the manufacture protocol.

### Western blot and antibodies

Western blot assay was performed as described before [21]. Cells were harvested and lysed using protein extraction reagent RIPA containing a protease inhibitor cocktail (Roche, Switzerland). About 25 μg proteins extraction were separated by SDS-PAGE, and were transferred to PVDF transfer membrane (Thermo Scientific, USA). After 1 h, the blots were incubated with primary antibodies to FGF2 (1:1000,) (Abcam, USA) and GAPDH (1:2500) (CST, USA) overnight. Then, the membranes were washed with PBS for three times and incubated with horseradish peroxidase-conjugated secondary antibody (1:1,000; Abcam) for 1 h. The antigen – antibody immunoreactivity was detected by Image Lab software (Bio-Rad, USA).

### Animal studies

In vivo xenografts studies were approved by the Animal Care and Use Committee. MG63 (1.0 × 10^7^) cells were injected subcutaneously into the flank of mice which were then incubated for 4 weeks (5 mice/group). When tumor volume approached 100 mm^3^, 100 µl miR-877-3p or empty vector lentiviruses (5 × 10^8^ TU/ml) were kept to inject intratumorally for three weeks. Tumor volumes were monitored routinely, and estimated by the formula: (width)^2^ × length/2. Finally, mice were sacrificed by cervical dislocation. Tumor tissues were collected for further tests .

### Statistical analysis

All data were expressed as mean ± standard deviation of three independent experiments. Statistical analysis was conducted with SPSS software package (version 16.0, SPSS Inc). Two-tailed Student’s t test or the Fisher LSD significance with one-way ANOVA test was used for parametric analyses. Mann-Whitney U test was used for nonparametric analyses. *P* < 0.05 was considered statistically significant.

## Results

In this study, we analyzed public available data in Gene Expression Omnibus (GEO, GSE65071), and further explored the top 10 downregulated microRNAs in GSE65071. We confirmed that miR‐877-3p expression was decreased in our OS samples in comparison with the normal counterparts. Moreover, miR‐877-3p overexpression could attenuate the proliferation in OS. We also found that miR‐877-3p could regulate angiogenesis *in vitro*. Angiogenesis ability of endothelial cells was impaired when miR‐877-3p was overexpressed in HUVEC. We found that miR‐877-3p decreased FGF2 expression through directly binding of FGF2 gene 3ʹ-UTR region to play antiproliferation and antiangiogenesis effects. So, we demonstrated that miR‐877-3p might serve as a novel therapeutic target of OS.

### miR-877-3p expression was decreased in OS

**Figure 1.**
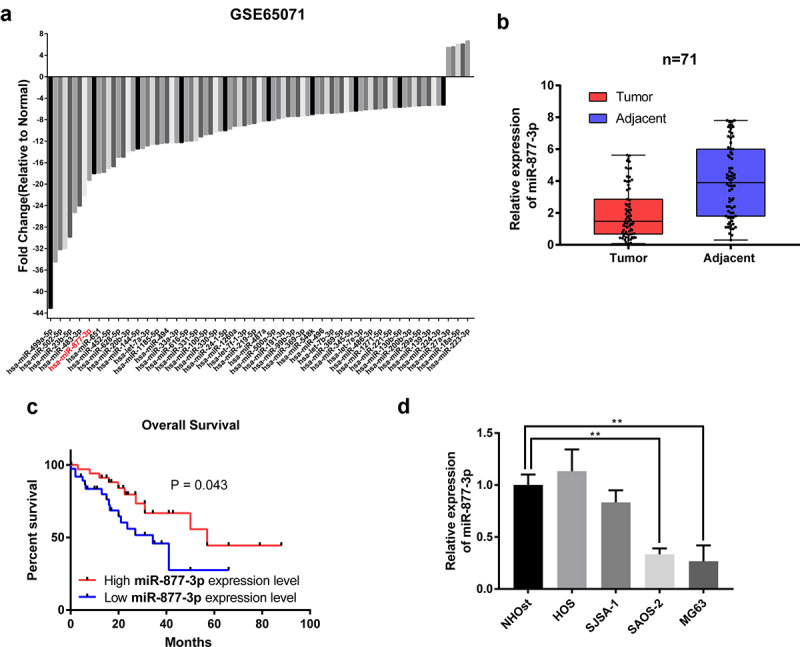
The expression of miR-877-3p in osteosarcoma. (a) In silico analysis using GEO database data GSE65071, including 20 human osteosarcoma tumor tissue samples and 15 control tissue samples. Differentially expressed miRNAs were plotted. (b) The expression of miR-877-3p in 71 samples of OS tissues and adjacent normal tissues were determined by real-time PCR. (c) The mRNA levels of miR-877-3p in OS tissues and adjacent normal tissues determined by real-time PCR. (d) overall survival of osteosarcoma patients based on miR-877-3p expression. (e) Expression level of miR-877-3p in osteosarcoma cells and normal osteoblast cell.

In silico analysis was performed using GEO data (GSE65071), including 20 human OS tumor and 15 control tissues. There were totally 87 miRNAs which were identified to differentially express between two groups, with the cutoff point of ≤0.5-fold changes for downregulated miRNAs and ≥2-fold changes for upregulated miRNAs. Hereinto, there were 5 upregulated microRNAs and 82 downregulated microRNAs (Figure 1(a)). Gene expression of top 10 downregulated microRNAs in GSE65071 were further investigated between 71 OS tissues and normal counterparts, including hsa-miR-499a-5p, hsa-miR-375, hsa-miR-502-5p, hsa-miR-624-5p, hsa-miR-483-3p, hsa-miR-490-3p, hsa-miR-23b-5p, hsa-miR-183-5p, hsa-miR10b-5p, and hsa-miR-877-3p. Among them, miR-499a-5p, miR-23b-5p, miR-183-5p, miR-877-3p were significantly downregulated in OS tissues compared to the adjacent noncancerous tissues (Figure 1(b), Table 1). Intriguingly, there have been no studies to address the role of miR-877-3p in OS before. So, we chose miR-877-3p for further investigation in OS. Since miR-877-3p is downregulated in OS tissues, we further studied the correlation between miR-877-3p expression and survival outcome, and found statistically significant decreased overall survival (OS) in low-expression group (*P* = 0.043, Figure 1(c)). Similarly, miR-877-3p was also found down-regulated in MG-63 and Saos-2 cells compared to normal human osteoblast NHOst cells (Figure 1(d)). Thus, we chose MG-63 and Saos-2 for further experiments. To summary, these results suggested that miR-877-3p might function as tumor suppressor in OS.

**Table 1.** Expression levels of candidate miRNAs

	Expression level	*P*
hsa-miR-499a-5p	Highly expressed in normal tissues	<0.001
hsa-miR-375	NS	0.405
hsa-miR-502-5p	NS	0.072
hsa-miR-624-5p	Highly expressed in tumor tissues	<0.001
hsa-miR-483-3p	NS	0.451
hsa-miR-490-3p	NS	0.108
hsa-miR-23b-5p	NS	0.012
hsa-miR-183-5p	Highly expressed in normal tissues	<0.001
hsa-miR10b-5p	NS	0.082

### miR-877-3p overexpression inhibited OS cell proliferation

**Figure 2.**
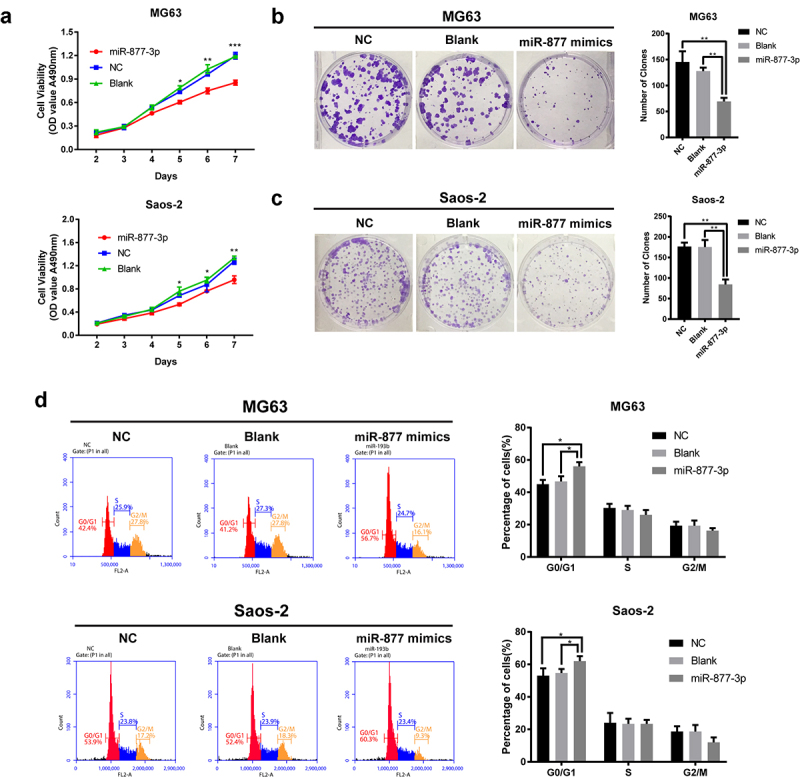
Effects of miR-877-3p on osteosarcoma proliferation. (a) After overexpression of miR-877-3p, MG63, and saos-2 cell proliferation was detected. Colony formation assay was performed to test the proliferation of (b) MG63 or (c) saos-2 cells. (d) Effect of miR-877-3p overexpression on cell cycle was measured by flow cytometry.

In order to investigate the role of miR-877-3p in tumor growth, we transfected MG-63 or Saos-2 cells with miR-877-3p mimics to figure out the actual effects of miR-877-3p on cell proliferation. Cell viability assay suggested that miR-877-3p overexpression remarkably inhibited OS cell proliferation at different time points (Figure 2(a)). Meanwhile, miR-877-3p mimics weakened cell clonal formation ability (Figures 2(b-c)). Flow cytometry was conducted to investigate the role of miR-877-3p overexpression in cell cycle distribution. As shown in (Figure 2(d)), miR-877-3p significantly increased the percentage of cells at G0/G1 phase in MG-63 and Saos-2 cells, indicating G0/G1 phase arrest of OS cells via miR-877-3p overexpression. Taken together, these data indicated that miR-877-3p suppressed proliferation of OS cells.

### miR-877-3p modulated angiogenesis activity of endothelial cells

**Figure 3.**
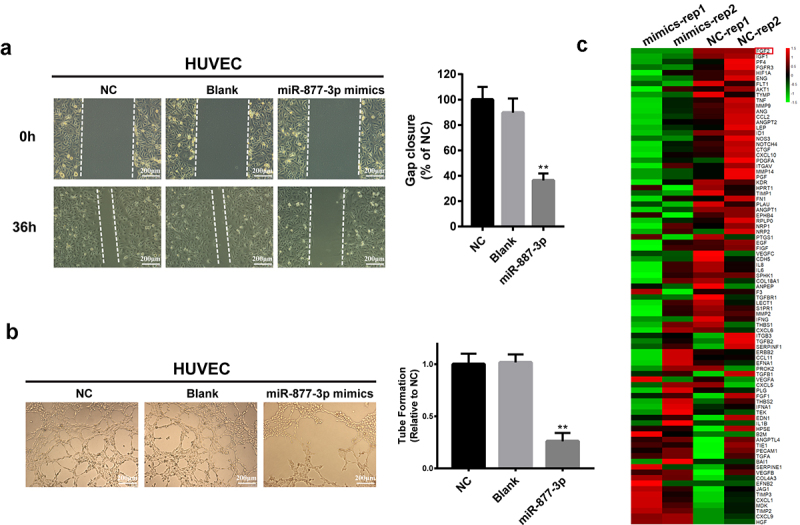
Effects of miR-877-3p on HUVECs. (a) Migration of transfected HUVECs in a scratch-wound assay 36 h after transfection. (b) Tube formation assay in HUVECs. (c) Heatmap of 84 angiogenesis genes in HUVECs.

To address the importance of miR-877-3p in antiangiogenesis therapy, HUVECs were transfected with miR-877-3p mimics or control oligos or lipo control. As shown in (Figure 3(a)), upregulated miR-877-3p significantly inhibited wound healing ability of HUVEC cells. Then, to further evaluate the effect of miR-877-3p on the angiogenic potential in HUVECs, tube formation assay was performed and the results showed that HUVECs transfected with miR-877-3p mimics significantly reduced number of tubes compared to controls (Figure 3(b)). Interestingly, miR-877-3p had no significant effects on HUVEC proliferation (data not shown). To gain insight into potential mechanisms of anti-angiogenesis by miR-877-3p, we profiled the expression genes involved in the biological processes of angiogenesis by using Human Angiogenesis RT^2^ Profiler PCR Array in HUVECs and found that FGF2 was the most downregulated gene among the 84 key angiogenesis related genes (Figure 3(c)). FGF2 is a key member of the fibroblast growth factor (FGF) family and has been reported to play an important role in tumor progression and angiogenesis. So, we speculated that miR-877-3p might exert its tumor suppression role by interacting with FGF2. These results suggested that miR-877-3p exerted antiangiogenesis effect through regulating FGF2 signaling.

### miR-877-3p exerted the antiproliferation and antiangiogenesis effects through suppressing FGF2

**Figure 4.**
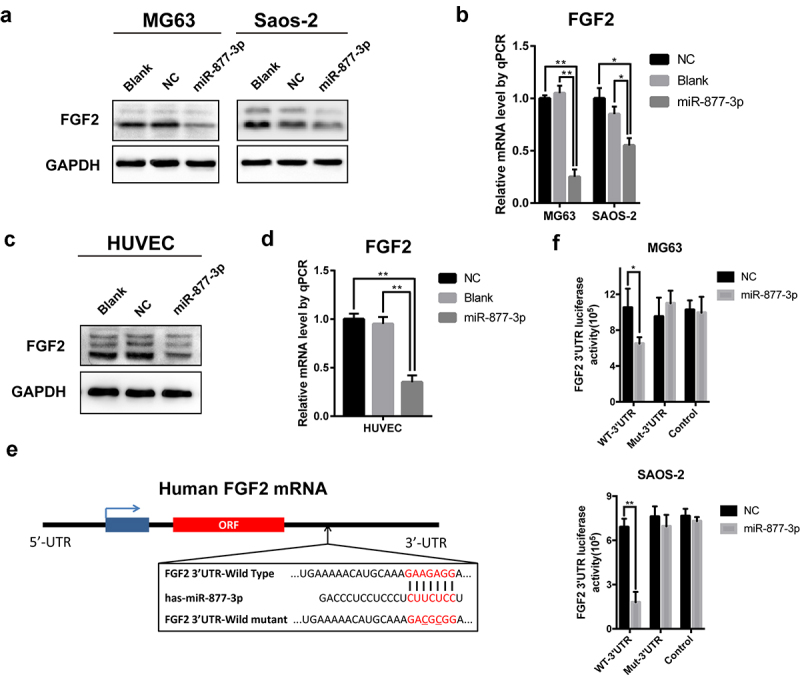
The relationship of miR-877-3p and FGF2 in osteosarcoma and HUVEC cells. After transfecting miR-877-3p mimics/control in MG63 and saos-2 cells. (a) The protein level of FGF2 was detected by Western blot analysis, (b) and the mRNA level of FGF2 was detected by real-time PCR. (c) The protein level and (d) the mRNA level of FGF2 were detected in HUVEC when transfected with miR-877-3p. (e) MiRDb predicted that miR-877-3p could target FGF2. (f) The interaction between miR-877-3p and FGF2 was examined by luciferase reporter assays.

Based on the results of PCR array, FGF2 was speculated to be the potential target of miR-877-3p. We then tested FGF2 expression in OS cell lines and HUVECs and found that miR-877-3p overexpression reduced FGF2 at transcriptional level, indicating that FGF2 might be a direct target of miR-877-3p (Figure 4(a-d)). We further sought to illustrate underlying molecular mechanism by which miR-877-3p suppressed FGF2 expression. MIRDb (http://www.mirdb.org/miRDb/) predicted that 3ʹ-UTR of FGF2 had target sequence for miR-877-3p (Figure 4(e)). Then, luciferase reporter vectors containing wild-type or mutant miR-877-3p target sequences of the FGF2 3ʹ- UTR were constructed. The luciferase reporter assay demonstrated that miR-877-3p upregulation inhibited the luciferase activity of the wild-type 3ʹ-UTR reporter gene (figure 4(f)), indicating that miR-877-3p can bind to the 3ʹ- UTR of FGF2.

**Figure 5.**
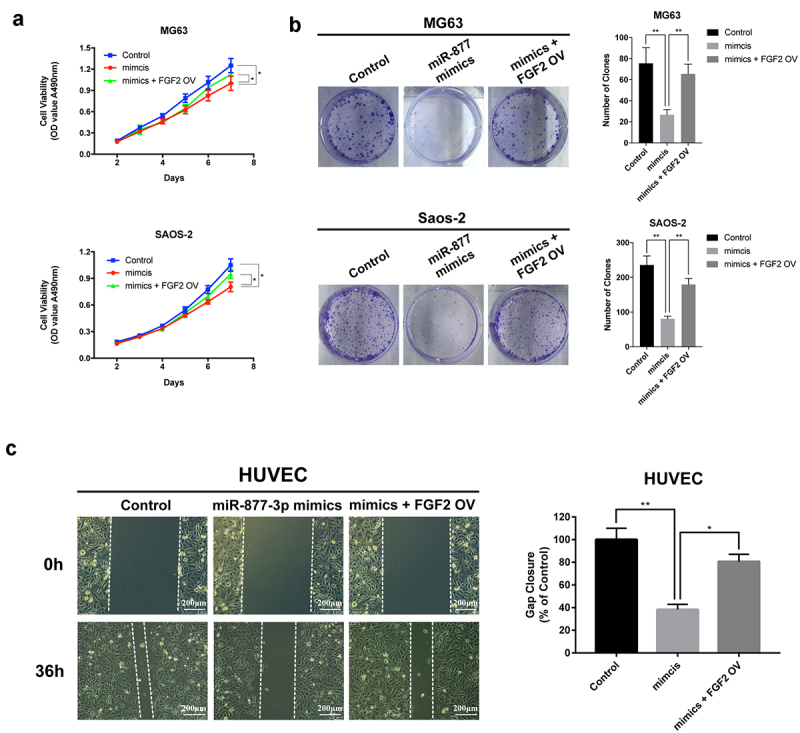
The effect of FGF2 overexpression in osteosarcoma and HUVEC cells. FGF2 was overexpressed in MG63 and saos-2 cells, cell proliferation was detected (a) by MTT assay and (b) colony formation assays. (c) HUVEC cells were transfected with control vector or miR-877-3p mimics only or both miR-877-3p mimics and FGF2 vectors, transfected HUVECs migration was detected in wound healing assay 36 h after transfection.

To confirm whether miR-877-3p suppresses tumor growth and angiogenesis through targeting FGF2, FGF2 was ectopically expressed using the overexpression plasmid (FGF2 OV). MTT, clonogenic and wound healing assays were conducted to assess the proliferation and angiogenesis and revealed that FGF2 restoration rescued the miR-877-3p effects on OS cells and HUVECs (Figure 5(a-c)). These results indicated that forced expression of miR-877-3p suppressed OS cell proliferation and angiogenesis through reducing FGF2 expression.

### miR-877-3p reduced OS cell growth and suppressed angiogenesis in vivo

**Figure 6.**
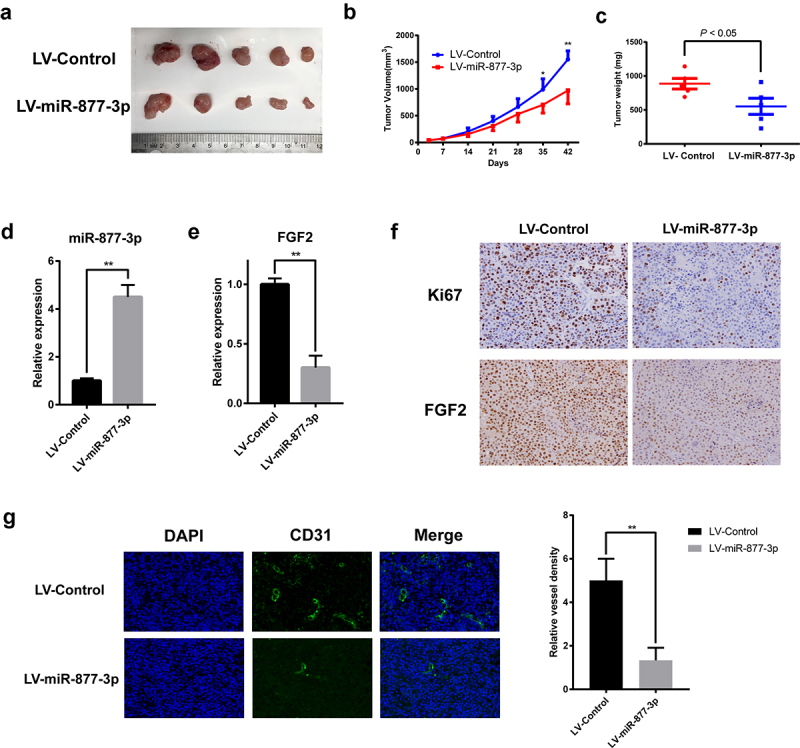
Effects of miR-877-3p on tumor growth and angiogenesis in mouse models. (a) MG63 tumors were harvested at the end of study, and (b) tumor volume and (c) tumor weight were measured. Expression level of miR-877-3p (d) and FGF2 (e) in tumor. (f) IHC staining of Ki67 and FGF2. (g) Immunofluorescence staining of CD31 for detection of blood vessels in MG63 tumor.

To determine the phenotype of miR-877-3p expression *in vivo*, we further investigated the effect of miR-877-3p on MG63 cell growth and angiogenesis in nude mice. We employed lentiviruses encoding miR-877-3p (LV-miR-877-3p) for *in vivo* delivery in a xenograft model. LV-miR-877-3p strongly suppressed tumor growth in MG63 tumor models compared with the vector group (LV-Control) (Figure 6(a-c)). Moreover, in LV-miR-877-3p group, decreased FGF2 expression and lower percentage of Ki67 positive cells were found, indicating that miR-877-3p could suppress tumor growth *in vivo* (Figure 6(d-f)). Next, the effect of miR-877-3p on tumor angiogenesis was investigated. Surprisingly, LV-miR-877-3p significantly inhibited tumor angiogenesis based on immunofluorescent staining of blood vessels by detecting PECAM (CD31) (Figure 6(g)). Together, these results suggested that miR-877-3p overexpression inhibited OS growth and angiogenesis *in vivo*.

## Discussion

OS is one of the most common primary malignant carcinoma a in bone tissues, characterized as rapid progress and poor prognosis in adolescence. The etiology of OS remains unclear, and metastasis occurs rapidly due to lack of diagnostic markers and therapeutic targets. Recent studies have suggested that genetic factors, such as miRNAs exhibited important regulatory functions in OS. In the present study identified significant role of miR-877-3p in OS. By targeting FGF2,miR-877-3p showed inhibitory effect on the proliferation of OS cell lines and angiogenesis of endothelial cell lines, thus suppress the development of OS.

Several past studies have investigated the role of miR-877-3p in various disease. Li et al. testified that miR-877-3p suppressed proliferation in bladder cancer by increasing the expression of tumor suppressor gene p16 [22]. In addition, miR-877-3p can act as suppressor for pulmonary fibrosis [23]. Moreover, several long noncoding RNA and circle RNA were reported sponging miR-877-3p and then derepressing the targeted mRNA by forming competing endogenous RNA (ceRNA) networks in various tumors such as non-small cell lung cancer, glioma, and cervical cancer [24–26]. The regulatory mechanism of miR-877-3p in OS remained largely unknown. In our study, we first discovered that miR-877-3p was under-expressed in OS tissues and most OS cell lines compared with normal counterparts. Low-expression of miR-877-3p predicted poor prognosis of OS patients, indicating that miR-877-3p may act as tumor suppressor in OS. Restoration of miR-877-3p in MG63 and Saos-2 cells significantly inhibited cell growth and induced G0/G1 arrest. Moreover, upregulation of miR-877-3p inhibited the growth of OS *in vivo*.

Tumor microenvironment consists of various kinds of cells including tumor cells, endothelial cells, fibroblasts, immune cells, and noncell components like extracellular matrix [27]. The growth of tumor mass and metastasis of tumor cell are dependent on the growth of local blood vessels. Antiangiogenesis has showed great effectiveness for treatment in several types of cancers including osteosarcoma. A phase II trial of VEGF receptor tyrosine kinase inhibitors (TKIs) sorafenib showed that high-grade OS patients can benefit from the antiangiogenesis therapy [28]. Recent researches have demonstrated that various miRNAs are involved in various physiological and pathological processes including angiogenesis. However, previous researches have never paid attention to the role of miR-877-3p in biological behaviors of endothelial cells. We sought to explore the effects of miR-877-3p overexpression on endothelial cells. Our results indicated that miR-877-3p overexpression inhibited of migration and tube formation, not proliferation, of HUVECs *in vitro*. In addition, we found the inhibitory effect of miR-877-3p on angiogenesis in OS *in vivo*. These results indicated that miR-877-3p might be a promising angiogenic related microRNA in OS.

Several regulatory factors have been recognized as stimulators of angiogenesis including activate hypoxia inducible factor (HIF-1α), vascular endothelial growth factor (VEGF), basic fibroblast growth factor (bFGF) and platelet derived growth factor (PDGF) [29]. One the other hand, a number of growth factors have showed direct or indirect interaction with miR-877-3p. A recent study demonstrated that miR-877 may act as a tumor suppressor by suppressing vascular endothelial growth factor A (VEGFA) in gastric cancer [30,31]. MiR-877 could also inhibit cell proliferation and invasion in non-small cell lung cancer by directly targeting IGF-1 R [32]. Besides, by silencing Smad7, miR-877-3p promoted osteoblastic differentiation of osteoblast precursor cell line induced by TGF-β1 [33]. According to PCR-array, FGF2 was the most significantly downregulated angiogenesis pathway genes upon miR-877-3p overexpression. Fibroblast growth factor receptor (FGFR) signaling has been reported to be implicated in both various biological processes and tumor development [34,35], including promoting the proliferation and angiogenesis abilities of cancer cells. FGF2 is the first proangiogenic factors identified in tumors, showing the direct effect on tumor angiogenesis. In our study, we found that FGF2 expression decreased in both OS cells and HUVECs, which was the most downregulated gene in miR-877-3p-overexpression HUVEC cell, suggesting that miR-877-3p might regulate FGF2 expression. Further, miR-877-3p regulated the angiogenesis and proliferation by binding to the 3ʹ-UTR of FGF2 mRNAs. Restoration of FGF2 expression in OS cells and HUVECs attenuated the anti-proliferation and antiangiogenesis effects of miR-877-3p, confirming the importance of miR-877-3p/FGF2 axis in inhibiting the growth and angiogenesis in OS cells.

Limitations existed in the present work. The sample size of OS patients is relatively limited to establish the relationship between the clinicopathological characteristics of OS patients and miR-877-3p expression. In addition, the detailed mechanism of FGF2 regulating proliferation of OS cell lines and angiogenesis of endothelial cells remains unclear. Further evaluation targeting FGF2 pathway on tumor development and tumor microenvironment is required.

## Conclusion

**Figure 7.**
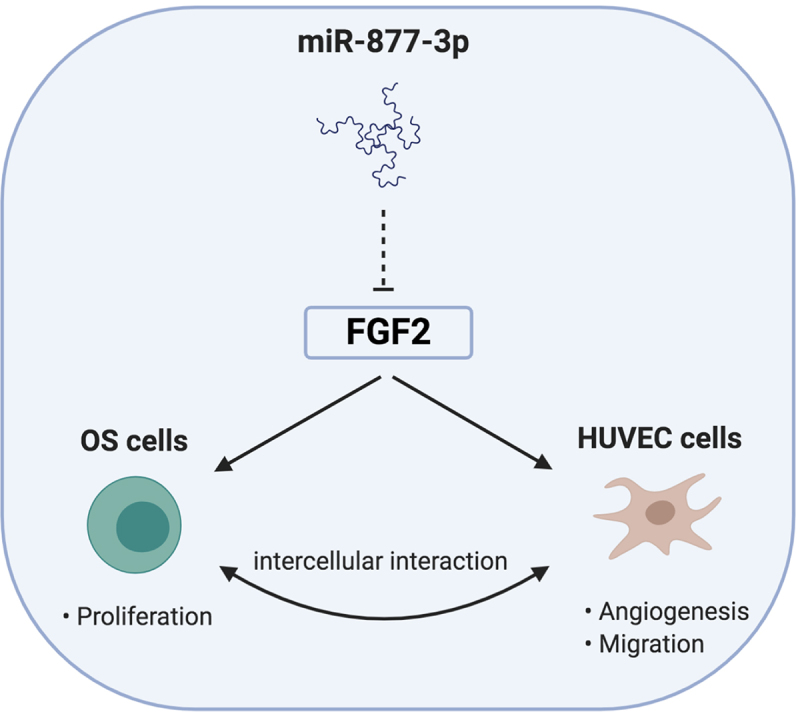
Schematic diagram of the proposed mechanism of miR-877-3p in OS. miR-877-3p could suppress proliferation of osteosarcoma cells and exert antiangiogenesis effect on HUVEC cells by targeting FGF2. As a result, the interactions of the OS cells and endothelial cells were interrupted.

Taken together, our study provides a new finding of miR-877-3p in OS and gives a noble view on the link between FGF2 and miR-877-3p. The expression of miR-877-3p was downregulated in OS tissues. OS patients with low miR-877-3p expression usually have a poor prognosis. By targeting the 3ʹ-UTR region of FGF2,miR-877-3p could act as a suppressor on the proliferation of OS cell lines and exert antiangiogenesis effect on the tumor microenvironment of OS (Figure 7). Our findings may provide a new potential target for the clinical diagnosis and treatment of OS in the future.

## Data Availability

The data used to support the findings of this study are available from the corresponding author upon request. The public data used in this study is available at http://www.ncbi.nlm.nih.gov/geo/

## HIGHLIGHTS

- miR-877-3p is underexpressed in osteosarcoma cell lines and tissues as compared to non-cancerous controls.
- miR-877-3p overexpression inhibits proliferation and mono-cloning formation of OS cells lines.
- miR-877-3p overexpression mitigates migration and angiogenesis of endothelial cell lines.
- miR-877-3p directly binds to the 3ʹ UTR region of tumor-promoting FGF2.
